# Molecular dynamics simulation of Sunset Yellow dye removal from water using hydrochar adsorbent

**DOI:** 10.1038/s41598-025-27733-z

**Published:** 2025-12-17

**Authors:** Thi H. Ho, Khoa Van Le, Phuong Tuyet Nguyen, Linh Nguyen, Thuat T. Trinh

**Affiliations:** 1 Laboratory for Computational Physics, Institute for Computational Science and Artificial Intelligence, Van Lang University, 70000 Ho Chi Minh City, Vietnam; 2 Faculty of Mechanical, Electrical, and Computer Engineering, Van Lang School of Technology, Van Lang University, Ho Chi Minh City, 70000 Vietnam; 3 Department of Chemistry, Faculty of Science, Tokyo University of Science, 1-3 Kagurazaka, Shinjuku-ku, 162-8601 Tokyo, Japan; 4 Faculty of Chemistry, University of Science, Vietnam National University-Ho Chi Minh City, Ho Chi Minh City, Vietnam; 5 Faculty of Interdisciplinary Science, University of Science, Ho Chi Minh City, Vietnam; 6 Division of Biomaterials and Tissue Engineering, Eastman Dental Institute, University College London, London, NW3 2PF UK; 7 Porelab, Department of Chemistry, Norwegian University of Science and Technology, NTNU, Trondheim, NO-7491 Norway

**Keywords:** Chemistry, Engineering, Environmental sciences, Materials science

## Abstract

Water pollution by synthetic dyes poses a serious environmental threat, necessitating effective and sustainable remediation technologies. This study explores the molecular mechanisms of Sunset Yellow (SSY) dye removal using hydrochar adsorbents via molecular dynamics simulations. Three hydrochar models were designed representing standard (with mixed functional groups), pure aromatic, and hydroxyl-enriched. All exhibited high SSY adsorption, with the standard model achieving adsorption capacities of 74–460 mg/g depending on SSY concentration, and 90% average efficiency. Adsorption increased with hydroxyl group density and decreased in their absence. Molecular analysis revealed that van der Waals interactions and $\pi$-$\pi$ stacking are the dominant mechanisms, with van der Waals forces being the strongest and scaling linearly with concentration. Hydroxyl functionalization enhanced adsorption by 35% compared to non-functionalized surfaces, while mixed functional groups offered the best balance of capacity and efficiency. The results demonstrate that functional group engineering critically influences adsorption performance, offering quantitative design principles for optimized hydrochar materials. These findings provide molecular-level insights to guide the development of advanced, sustainable adsorbents for water treatment.

## Introduction

Water pollution caused by synthetic dyes represents one of the most pressing environmental challenges of the 21 st century. The textile, food, pharmaceutical, and cosmetic industries discharge billions of liters of dye-contaminated wastewater annually, creating severe ecological and human health risks^1^. Among the various classes of synthetic dyes, azo dyes constitute approximately 70% of all commercial dyes due to their superior color fastness, ease of synthesis, and cost-effectiveness^2,3^. However, these same properties that make azo dyes industrially valuable also render them highly persistent environmental contaminants. Common dye removal methods such as coagulation-flocculation, membrane filtration, advanced oxidation, and biological degradation^1^ face limitations including high costs, incomplete mineralization, toxic byproducts, or stringent operational requirements^4^. Adsorption stands out due to its simplicity, cost-effectiveness, and regenerability, yet developing efficient, sustainable, and economically viable adsorbents remains a key challenge.

Sunset Yellow (SSY) exemplifies the dual nature of azo dye applications and environmental concerns^5^. Widely used as a food coloring agent and in textile manufacturing, Sunset Yellow exhibits excellent stability under normal processing conditions. The molecular structure of Sunset Yellow, characterized by its planar aromatic framework and azo linkage, exhibits unique self-assembly properties that have been extensively studied in liquid crystal systems^6^. These molecular ordering characteristics, while beneficial for certain applications, also contribute to the dye’s environmental persistence and complex adsorption behavior. Nevertheless, its release into aquatic systems poses significant threats due to its potential carcinogenic and mutagenic properties, particularly following metabolic breakdown to aromatic amines^7^. The high water solubility of Sunset Yellow and its anionic nature at neutral pH further complicate removal efforts, as conventional biological treatment systems often prove ineffective against such recalcitrant compounds^8^.

In recent years, biochar and hydrochar have attracted considerable attention as sustainable adsorbents for environmental remediation applications^9^. Hydrochar, produced through hydrothermal carbonization (HTC) of biomass at moderate temperatures (180–250$^{\circ }$C) in the presence of water, represents a particularly promising alternative to conventional adsorbents. Recent molecular dynamics studies have provided detailed insights into the HTC process, revealing the complex reaction mechanisms and structural transformations that occur during hydrochar formation^10^. Unlike pyrolytic biochar, hydrochar retains beneficial functional groups including carboxyl, hydroxyl, and carbonyl moieties that enhance adsorption capacity through multiple interaction mechanisms^11^. The HTC process also allows for precise control over surface chemistry and porosity, enabling the design of application-specific adsorbents with optimized properties.

The complex nature of dye-adsorbent interactions necessitates a fundamental understanding of the underlying molecular mechanisms governing adsorption processes. Recently, Petrović et al.^12^ reported complementary insights into the effects of hydroxyl and aromatic functional groups of hydrochar on adsorption efficiency and stability. While experimental studies provide valuable macroscopic insights into adsorption kinetics, isotherms, and thermodynamics, they offer limited information about the molecular-level events that determine adsorption selectivity and capacity^13^. Molecular dynamics (MD) simulations have emerged as powerful computational tools for investigating these microscopic phenomena, providing atomic-level details about adsorbate-adsorbent interactions, surface binding mechanisms, and the role of solvent molecules in adsorption processes^14,15^. Recent theoretical studies have demonstrated the capability of MD simulations and density functional theory to elucidate water adsorption mechanisms on various material surfaces, providing valuable insights into the molecular-level understanding of adsorption processes^16^. Furthermore, advanced MD studies of hydrogen adsorption on graphitic surfaces have shown excellent agreement between computational predictions and experimental thermodynamic properties, validating the accuracy of molecular simulation approaches for adsorption systems^17^. Recent advances in reactive force fields, such as ReaxFF, have enabled the accurate simulation of complex chemical processes in aqueous environments, including environmental remediation applications for organic pollutants and microplastics^18–20^.

Despite the growing body of research on dye adsorption and the recognized potential of hydrochar as a sustainable adsorbent, several critical knowledge gaps remain that limit our ability to rationally design optimal adsorbent materials. First, the molecular-level mechanisms governing dye-hydrochar interactions are poorly understood, with most studies focusing on macroscopic adsorption behavior rather than fundamental molecular processes. Second, the role of specific functional groups in determining adsorption selectivity and capacity has not been systematically investigated at the atomic level. Third, the influence of surface chemistry on adsorption kinetics, binding configurations, and competitive effects remains largely unexplored. These knowledge gaps are particularly significant for practical water treatment applications, where understanding the molecular basis of adsorption can inform the design of more efficient, selective, and cost-effective adsorbent materials. The ability to predict adsorption performance based on molecular structure would enable the development of tailored hydrochar materials optimized for specific pollutants and operating conditions, reducing the need for extensive experimental screening and optimization.

The present study addresses critical research gaps by employing comprehensive molecular dynamics simulations to investigate Sunset Yellow adsorption on hydrochar surfaces. This study employs molecular dynamics simulations to bridge the gap between macroscopic adsorption performance and molecular-scale interaction mechanisms, offering dynamic, atomistic insights that are inaccessible through experimental methods alone. The novelty of this study lies in the systematic molecular-level investigation of functional group effects on hydrochar using tailored models in molecular dynamics simulations, providing quantitative design principles for optimized dye adsorption that have not been previously established. Through systematic computational analysis, we aim to elucidate molecular interactions responsible for dye binding, quantify the role of specific functional groups in determining adsorption performance, and establish quantitative relationships between surface chemistry and adsorption capacity. By showcasing hydrochar’s exceptional potential as a sustainable and efficient dye adsorbent material, our findings will contribute significantly to the development of advanced wastewater treatment technologies. These fundamental insights can also be extended to other pollutant-adsorbent systems, helping address global water pollution challenges.

## Methods

### Molecular model

The structure and dewaterability of hydrochar are influenced by both the composition of the biomass used and the specific reaction conditions applied during HTC process^21^. Recent molecular simulation studies have demonstrated that chemical functional groups play a crucial role in determining hydrochar properties and interactions^22^. In this study, the initial structure of hydrochar was adopted based on experimental observations from the HTC of glucose^23^. Given that glucose serves as a widely utilized model compound in the examination of HTC biomass, its selection was motivated by its prevalence in previous studies^23,24^.

**Fig. 1:**
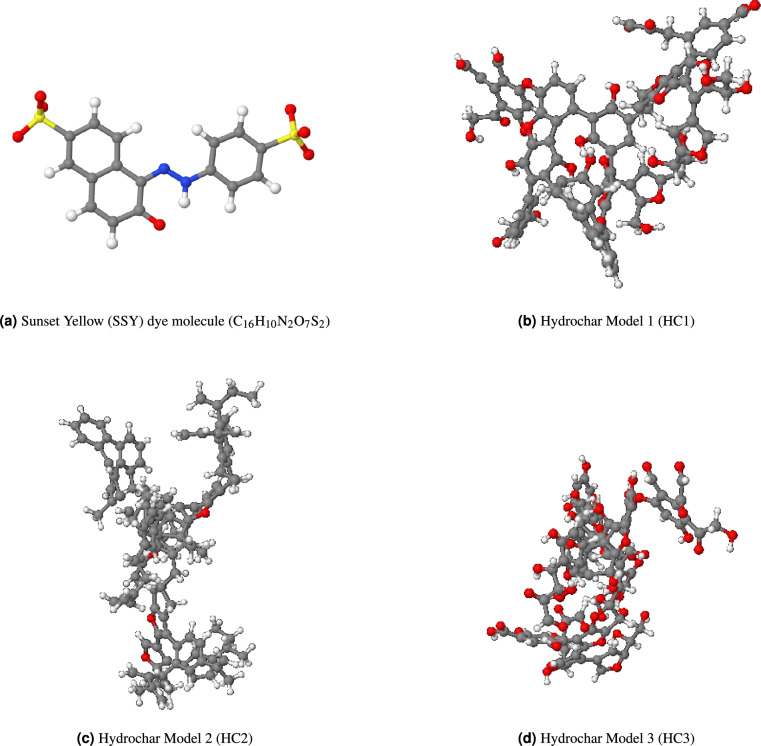
Molecular structures used in the simulation study. (**a**) Sunset Yellow dye. Hydrochar variants: (**b**) HC1 (mixed functional groups), (**c**) HC2 (purified carbon framework for $\pi$-$\pi$ interactions), (**d**) HC3 (hydroxyl-enriched). Atom colors: C (grey), H (white), O (red), N (blue), S (yellow).

Three distinct molecular models of hydrochar were constructed to systematically deconvolute the contribution of specific functional groups to dye adsorption mechanisms (Fig. 1). The baseline model, HC1 (Mixed-FG), represents a realistic structure based on experimental characterization of glucose-derived hydrochar, incorporating a typical distribution of oxygen-containing functional groups, including hydroxyl (-OH), carboxyl (-COOH), and carbonyl (C=O). To isolate the mechanism of non-specific interactions, model HC2 (Carbon-Only) was derived from HC1 by removing all functional groups, leaving a purely aromatic carbon framework. This model specifically targets the analysis of $\pi$-$\pi$ stacking and hydrophobic effects. Conversely, model HC3 (Hydroxyl-Rich) was designed as a functionalized variant of HC1 with a strategically increased density of hydroxyl groups to probe the specific effects of enhanced hydrogen bonding capacity and surface polarity.

The functional group composition of each hydrochar model was systematically quantified through detailed analysis of the molecular structures. Table 1 presents the comprehensive functional group inventory for all three hydrochar architectures, providing quantitative insights into the surface chemistry differences that govern adsorption behavior.

**Table 1:** Functional group quantification and characterization for three hydrochar models.

Molecule	Formula	O/C ratio	$\hbox {n}_{\textrm{OH}}$ (hydroxyl)	$\hbox {n}_{\textrm{COOH}}$ (carboxyl)	$\hbox {n}_{\textrm{CO}}$ (ketone, ether)
[HC1 (Mixed-FG)	$\hbox {C}_{99}\hbox {H}_{70}\hbox {O}_{33}$	0.33	13	1	18
HC2 (Carbon-only)	$\hbox {C}_{128}\hbox {H}_{130}\hbox {O}_{4}$	0.03	0	0	4
HC3 (Hydroxyl-Rich)	$\hbox {C}_{99}\hbox {H}_{72}\hbox {O}_{54}$	0.55	33	2	17

Table 2 provides a comprehensive overview of all simulation systems constructed for this study, including the system composition and box dimensions for each hydrochar type and SSY concentration. The table demonstrates the systematic variation in system size and composition, accommodating the increasing number of dye molecules while maintaining appropriate density and solvation conditions.

**Table 2:** Simulation system specifications for different hydrochar models. Each system contains 100 hydrochar molecules with varying numbers of SSY dye molecules (10–300) in water. Values shown are ranges across all SSY concentrations. A total of 42 simulation systems were constructed and analyzed.

HC Type	SSY range	HC count	Water molecules	Total Atoms
HC1	10–300	100	42,185–69,085	147,145–239,155
HC2	10–300	100	40,577–67,619	148,121–240,557
HC3	10–300	100	41,877–68,911	148,321–240,733

### Methods

All molecular dynamics simulations were performed using GROMACS version 2024.4^25^. The General Amber Force Field 2 (GAFF2)^26^ was employed to describe the intermolecular interactions for both hydrochar and Sunset Yellow dye molecules. This force field has been previously validated for molecular simulations of Sunset Yellow dye in liquid crystal systems, demonstrating its accuracy in capturing the molecular ordering and self-assembly properties of this azo dye^27^. Force field parameters were assigned using the ANTECHAMBER tools for automatic atom type and bond type perception^28^. Water molecules were modeled using the SPC/E water model, which provides accurate representation of water properties including density, diffusion coefficients, and dielectric constant^29^.

The simulation system was constructed in a cubic simulation box with periodic boundary conditions applied in all three dimensions. Temperature was maintained at 298.15 K using the velocity-rescaling thermostat (V-rescale)^30^ with a coupling time constant of 0.1 ps. Pressure was controlled at 1 bar using the Parrinello-Rahman barostat^31^ with a coupling time constant of 2.0 ps and compressibility of $4.5 \times 10^{-5}$ bar$^{-1}$. Long-range electrostatic interactions were calculated using the Particle Mesh Ewald (PME) method^32^ with a real-space cutoff of 1.0 nm, while van der Waals interactions were truncated at 1.0 nm with long-range dispersion corrections applied.

**Fig. 2:**
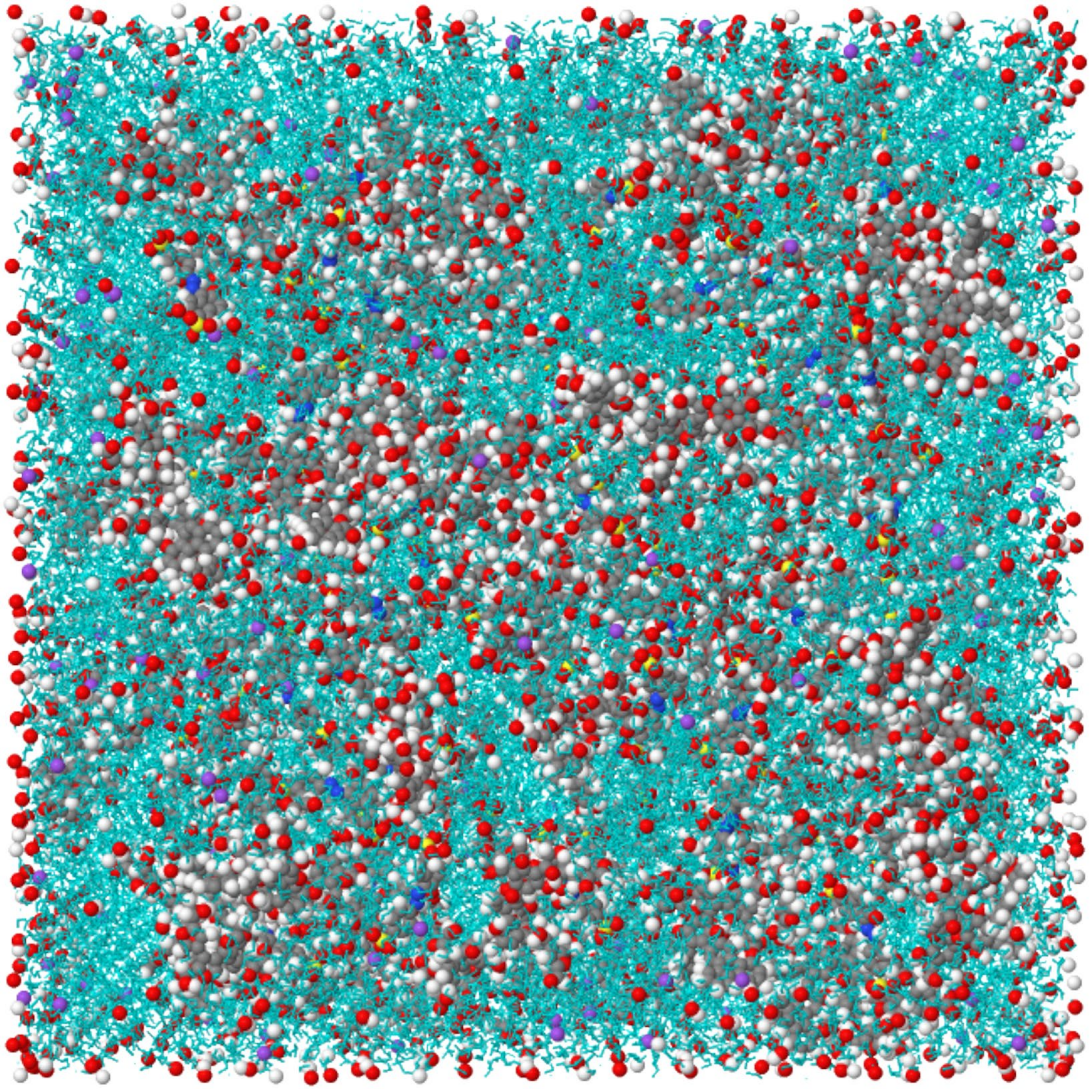
Representative snapshot of the initial solvated simulation system containing 100 hydrochar (HC1) molecules and 100 Sunset Yellow dye molecules in water. The system demonstrates the random initial distribution of components before equilibration and production runs. Atoms are colored by element: C (grey), H (white), O (red), N (blue), and S (yellow). Water is presented in stick model with green color.

Each simulation system was constructed by placing 100 hydrochar molecules in a cubic simulation box, followed by the addition of SSY dye molecules at varying concentrations spanning 10 to 300 molecules. The system was then solvated with SPC/E water molecules to achieve a box density consistent with experimental conditions. Sodium ions (Na$^{+}$) are included as part of the SSY disodium salt in aqueous solution; no additional counterions were added. Figure 2 shows a representative snapshot of the initial solvated system containing 100 HC1 molecules and 100 SSY dye molecules in water.

The molecular dynamics simulation protocol followed a sequential energy minimization and equilibration procedure prior to production runs. Initial system configurations were generated by randomly placing dye molecules at least 0.5 nm from the hydrochar surface to prevent initial steric clashes. Energy minimization was then performed to remove high-energy contacts, beginning with a steepest descent algorithm until the maximum force fell below 1000 kJ mol$^{-1}$ nm$^{-1}$, followed by a conjugate gradient method converging to a stricter tolerance of 100 kJ mol$^{-1}$ nm$^{-1}$. Subsequently, the system was equilibrated in two phases: first, a 1 ns NVT ensemble simulation with position restraints applied to hydrochar heavy atoms to relax the solvent and dye molecules around the fixed adsorbent; and second, a 2 ns NPT ensemble simulation with all restraints released to achieve proper system density and pressure. Finally, a 20 ns production simulation was conducted in the NPT ensemble using a 2 fs time step. Throughout this phase, coordinates were saved every 10 ps for trajectory analysis, and energies were recorded every 1 ps. All bonds involving hydrogen were constrained using the LINCS algorithm^33^ to enable the use of this larger integration time step.

### Analysis methods

Following molecular dynamics simulations, a comprehensive analysis of the adsorption behavior was conducted using multiple computational approaches. The adsorption kinetics were quantified by monitoring the number of dye molecules within a specific distance cutoff from the hydrochar surface as a function of simulation time. Adsorption isotherms were subsequently constructed by plotting the equilibrium number of adsorbed dye molecules against the initial bulk concentration for each simulated system.

To elucidate intermolecular interactions, radial distribution functions (RDFs) were computed using the native analysis tools within GROMACS. Furthermore, hydrogen bonding interactions between dye molecules and hydrochar functional groups were quantified by applying standard geometric criteria: a donor-acceptor distance of less than 0.35 nm and a donor-hydrogen-acceptor angle greater than 150$^{\circ }$.

The adsorption isotherm analysis provides quantitative insights into the equilibrium adsorption behavior and allows for the determination of key thermodynamic parameters. Following the molecular dynamics simulations, adsorption isotherms were constructed by plotting the equilibrium adsorption capacity ($Q_e$) against the equilibrium concentration ($C_e$) for each hydrochar system across the full range of SSY concentrations.

The equilibrium adsorption capacity ($Q_e$) was calculated as the average number of adsorbed SSY molecules per hydrochar molecule over the last 50% of the simulation trajectory:

1 $$\begin{aligned} Q_e = \frac{N_{ads}}{N_{HC}} \end{aligned}$$

where $N_{ads}$ is the number of adsorbed SSY molecules and $N_{HC}$ is the number of hydrochar molecules.

The equilibrium concentration ($C_e$) was determined from the number of SSY molecules remaining in solution:

2 $$\begin{aligned} C_e = \frac{N_{sol}}{V_{box} \cdot N_A} \end{aligned}$$

where $N_{sol}$ is the number of SSY molecules in solution, $V_{box}$ is the simulation box volume, and $N_A$ is Avogadro’s number.

The adsorption efficiency (*E*) was calculated as the percentage of total SSY molecules that were adsorbed:

3 $$\begin{aligned} E = \frac{N_{ads}}{N_{total}} \times 100\% \end{aligned}$$

where $N_{total}$ is the total number of SSY molecules in the system.

The simulation adsorption data were fitted to both Langmuir and Freundlich isotherm models to understand the underlying adsorption mechanisms and quantify thermodynamic parameters.

**Langmuir Isotherm Model:** The Langmuir model assumes monolayer adsorption on a homogeneous surface with equivalent adsorption sites and no interaction between adsorbed molecules^34^:

4 $$\begin{aligned} Q_e = \frac{Q_{max} \cdot K_L \cdot C_e}{1 + K_L \cdot C_e} \end{aligned}$$

where:

- $Q_e$ is the equilibrium adsorption capacity (mol/mol)
- $Q_{max}$ is the maximum adsorption capacity (mol/mol)
- $K_L$ is the Langmuir constant (L/mol) related to adsorption affinity
- $C_e$ is the equilibrium concentration (mol/L)

The linearized form of the Langmuir equation is:

5 $$\begin{aligned} \frac{C_e}{Q_e} = \frac{1}{Q_{max} \cdot K_L} + \frac{C_e}{Q_{max}} \end{aligned}$$

**Freundlich Isotherm Model:** The Freundlich model describes multilayer adsorption on heterogeneous surfaces with varying adsorption energies^35^:

6 $$\begin{aligned} Q_e = K_F \cdot C_e^{1/n} \end{aligned}$$

where:

- $K_F$ is the Freundlich constant [(mol/mol)(L/mol)$^{1/n}$] indicating adsorption capacity
- *n* is the Freundlich exponent indicating adsorption intensity

The linearized form of the Freundlich equation is:

7 $$\begin{aligned} \ln Q_e = \ln K_F + \frac{1}{n} \ln C_e \end{aligned}$$

The quality of fit for each isotherm model was evaluated using the coefficient of determination ($R^2$):

8 $$\begin{aligned} R^2 = 1 - \frac{\sum _{i=1}^{n} (Q_{e,\textrm{sim},i} - Q_{e,\textrm{calc},i})^2}{\sum _{i=1}^{n} (Q_{e,\textrm{sim},i} - \bar{Q}_{e,\textrm{sim}})^2} \end{aligned}$$

where $Q_{e,\textrm{sim},i}$ and $Q_{e,\textrm{calc},i}$ are the simulated and calculated adsorption capacities, respectively, and $\bar{Q}_{e,\textrm{sim}}$ is the mean simulated value.

To understand the stability of adsorption performance across different operating conditions, concentration-dependent efficiency analysis was performed. The efficiency change ($\Delta E$) between low and high concentration regimes was calculated:

9 $$\begin{aligned} \Delta E = E_{high} - E_{low} \end{aligned}$$

where $E_{high}$ and $E_{low}$ represent the average efficiency in the high ($\ge$200 SSY) and low ($\le$50 SSY) concentration ranges, respectively.

It is important to note that this study is entirely computational, and no experimental validation was performed. All findings are based on molecular dynamics simulations and theoretical analyses.

## Results and discussion

### Molecular-level interactions and water structure

The molecular-level interactions in the hydrochar-dye system were characterized through radial distribution function (RDF), providing fundamental insights into water structure and solvation effects. The RDF provides insight into the local structural organization around specific atom pairs by measuring the probability of finding atoms at a given distance. Peaks in the RDF correspond to preferred interatomic distances and indicate strong non-covalent interactions. However, RDF analysis reflects time-averaged spatial correlations and does not directly confirm bond formation or dynamics. Two complementary RDF analyses reveal the structural basis of the adsorption mechanism and the role of water in the system.

**Fig. 3:**
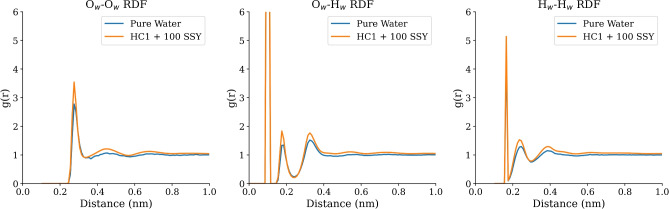
Comparison of water radial distribution functions (RDFs) between pure water and HC1+100 SSY systems. (**a**) O$_w$-O$_w$ RDF showing water oxygen-water oxygen interactions, (**b**) O$_w$-H$_w$ RDF showing water oxygen-water hydrogen interactions, and (**c**) H$_w$-H$_w$ RDF showing water hydrogen-water hydrogen interactions.

Analysis of the aqueous environment reveals that the presence of hydrochar and dye molecules induces minor perturbations in local water structure. Figure 3 provides a comparative RDF of three fundamental water-water interactions in pure water versus the HC1+100 SSY system. The O$_w$-O$_w$ RDF (panel a) demonstrates a more intense first solvation shell peak compared to pure water. This enhanced structuring indicates that the hydrochar surface templates a more ordered, hydration layer, which has important implications for the solvation forces mediating adsorption. The O$_w$-H$_w$ RDF (panel b), which probes intramolecular geometry and hydrogen bonding, shows a slight shift in the first peak, indicating that the presence of the adsorbent modifies the preferred orientation of water molecules in the interfacial region. Finally, the H$_w$-H$_w$ RDF (panel c) provides information on the hydrogen-hydrogen correlations, revealing a restructuring of the broader hydrogen-bonding network around the hydrophobic domains of the hydrochar. Together, these changes in hydration patterns are critical for understanding the driving forces and energetics of the adsorption process.

**Fig. 4:**
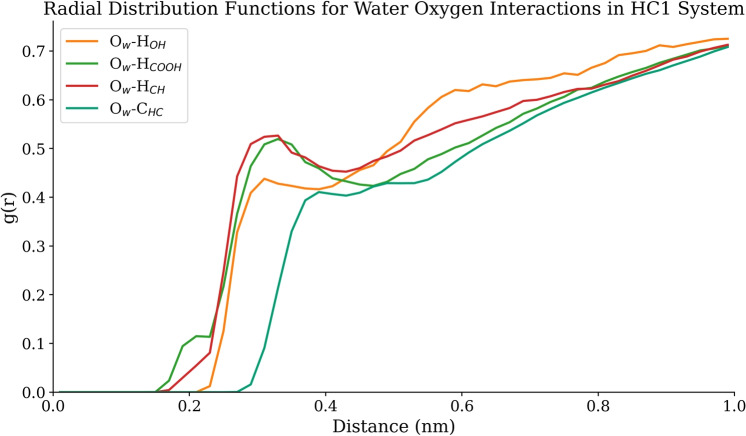
Radial distribution functions for water oxygen interactions with hydrochar functional groups in the HC1 system with 100 SSY molecules. The plot shows four key interactions: O$_w$-H$_{OH}$ (water oxygen-hydroxyl hydrogens), O$_w$-H$_{COOH}$ (water oxygen-carboxyl hydrogens), O$_w$-H$_{CH}$ (water oxygen-aliphatic/aromatic hydrogens), and O$_w$-C$_{HC}$ (water oxygen-hydrochar carbon).

Radial distribution functions (Fig. 4) quantify the distinct solvation environments around hydrochar functional groups. The RDF peaks for water interacting with hydrochar functional groups are inherently lower in intensity than those for the O$_w$-H$_w$ water-water interaction. This is a direct consequence of the vastly greater number of water-water pairs in the system compared to the number of specific functional group-water pairs available for sampling. The RDFs reveal distinct hydration distances that water oxygen (O$_w$) atoms are located at approximately 0.30 nm from hydrogen atoms of hydrochar functional groups, indicating direct hydration interactions. In contrast, the O$_w$-carbon (C$_{HC}$) distance is longer, around 0.40 nm, reflecting a weaker hydration shell around the hydrophobic carbon framework.

**Fig. 5:**
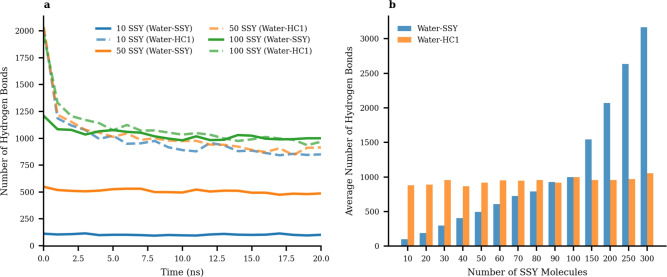
Hydrogen bond for Cbase hydrochar-dye systems. (**a**) Time evolution of hydrogen bonds for 10, 50, and 100 SSY systems showing water-SSY (solid lines) and water-HC1 (dashed lines) interactions over the 20 ns simulation period. (**b**) Average hydrogen bonds vs SSY concentration demonstrating the systematic relationship between dye loading and molecular interactions.

The temporal evolution of hydrogen bonding interactions during the simulation provides insights into the adsorption mechanism across different dye concentrations. Figure 5 presents a detailed picture of hydrogen bond formation between water molecules and both SSY dye molecules and HC1 hydrochar, revealing concentration-dependent interaction patterns and temporal dynamics.

A hydrogen bond analysis provides valuable insights into the molecular-level adsorption mechanism. Hydrogen bonds were identified based on geometric criteria as presented in the previous section. These definitions provide a practical framework for analyzing dominant interaction patterns but cannot capture the full quantum mechanical nature or short-lived hydrogen bonding events. Figure 5(a) presents the temporal evolution of hydrogen bonding for three representative systems (10, 50, and 100 SSY molecules), revealing distinct patterns that correlate with dye concentration. The time series highlights that all systems initially exhibit high hydrogen bonding, which gradually equilibrates over time. The rate and extent of change in hydrogen bonding are directly related to the number of dye molecules present, emphasizing the concentration-dependent nature of the adsorption process. All systems display rapid initial changes followed by stabilization. The 100 SSY system exhibits the most significant evolution, underscoring the increased complexity and dynamic nature of the adsorption mechanism at higher dye concentrations. Furthermore, the number of hydrogen bonds between HC1 and water decreases during the simulations, indicating that as SSY molecules are adsorbed onto HC1, water molecules surrounding the HC1 are replaced by SSY. This observation highlights the competitive nature of the adsorption process and the role of hydrogen bonding in mediating the interactions between dye, adsorbent, and solvent molecules.

The concentration-dependent behavior presented in Fig. 5(b) offers quantitative evidence of the systematic relationship between dye loading and hydrogen bonding patterns. The water-SSY hydrogen bonds exhibit a strong linear correlation with SSY concentration, increasing from approximately 102 bonds for 10 SSY molecules to 3,168 bonds for 300 SSY molecules. This linear scaling demonstrates that each additional dye molecule contributes proportionally to the total hydrogen bonding capacity, indicating efficient utilization of available binding sites and highlighting the concentration-dependent nature of the adsorption process. In contrast, water-HC1 hydrogen bonds remain relatively stable across all concentrations. This observation is mainly attributed to the constant number of HC1 in the system.

The competitive binding mechanism revealed by this analysis explains the high adsorption efficiency observed experimentally. As SSY concentration increases, dye molecules progressively displace water from hydrochar binding sites, leading to the formation of stable dye-hydrochar complexes. The fact that water-HC1 bonds remain relatively constant while water-SSY bonds scale linearly with concentration suggests that the adsorption process involves selective replacement of water molecules at specific interaction sites rather than complete surface coverage.

### Adsorption process and concentration dependence

**Fig. 6:**
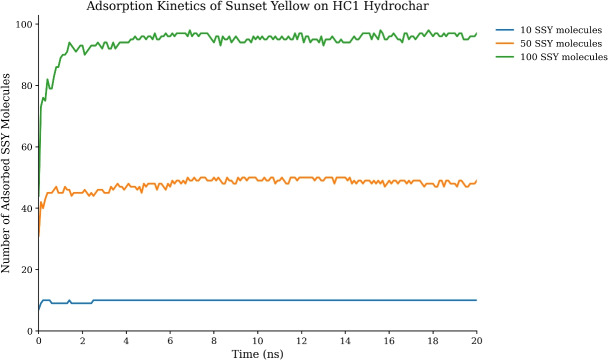
Concentration-dependent adsorption kinetics of Sunset Yellow (SSY) dye molecules onto HC1 hydrochar surfaces during the 20 ns molecular dynamics simulation. The plot shows the temporal evolution of adsorbed SSY molecules for three representative systems: 10 SSY, 50 SSY, and 100 SSY molecules. All systems exhibit rapid initial adsorption followed by equilibrium stabilization, with the final adsorption efficiency showing systematic dependence on dye concentration.

The adsorption behavior of Sunset Yellow dye molecules onto hydrochar surfaces was systematically analyzed across different dye concentrations to understand both the kinetics and concentration-dependent behavior of the removal process. Figure 6 presents the temporal evolution of the number of SSY molecules adsorbed onto HC1 hydrochar surfaces for three representative systems containing 10, 50, and 100 SSY molecules over the 20 ns simulation period.

The concentration-dependent adsorption reveals several key characteristics of the SSY-HC1 interaction system across different dye loadings. The most important feature is the rapid initial adsorption phase observed in all three systems, where the number of adsorbed dye molecules increases dramatically within the first 2–3 ns of simulation time. This rapid uptake demonstrates the strong thermodynamic driving force for dye-hydrochar association and indicates highly favorable binding interactions between the anionic dye molecules and the functionalized hydrochar surface.

The concentration-dependent behavior reveals important insights into the adsorption mechanism. For the 10 SSY system, adsorption reaches 100% efficiency rapidly and maintains this level throughout the simulation, indicating complete utilization of available binding sites. The 50 SSY system achieves approximately 98% adsorption efficiency, while the 100 SSY system reaches about 95% efficiency, demonstrating that higher dye concentrations lead to slightly lower final adsorption percentages due to competitive binding and steric effects. Following the initial rapid adsorption phase, all systems reach stable plateaus where the number of adsorbed molecules remains essentially constant throughout the remainder of the simulation. This equilibrium behavior indicates that the adsorption process has reached a steady state where the rates of adsorption and desorption are balanced. The high plateau values (95–100% adsorption) confirm the exceptional efficiency of HC1 hydrochar for SSY dye removal across different concentration ranges.

**Fig. 7:**
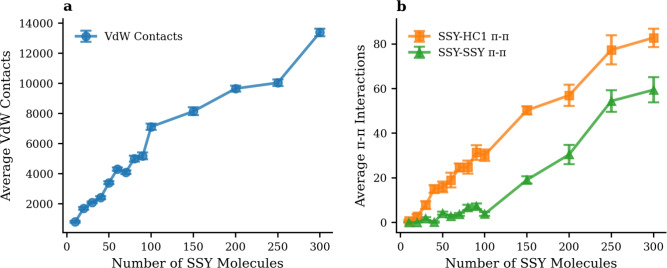
Concentration-dependent molecular interactions between SSY dye molecules and HC1 hydrochar across 14 different SSY loading systems. (**a**) Average van der Waals contacts. (**b**) Average $\pi$-$\pi$ stacking interactions demonstrating preferential SSY-HC1 binding over SSY-SSY self-aggregation.

To further understand the molecular basis of the strong SSY-HC1 interactions, the concentration-dependent behavior of specific intermolecular interactions was systematically analyzed across all 14 SSY concentration systems. Figure 7 presents van der Waals contacts and $\pi$-$\pi$ stacking interactions as a function of SSY concentration, revealing systematic patterns that govern the adsorption mechanism.

The van der Waals interaction (Fig. 7a) reveals that physical contacts between dye and hydrochar scale linearly from approximately 800 contacts for 10 SSY molecules to over 13,000 contacts for 300 SSY molecules. This strong linear relationship indicates that the hydrochar surface provides abundant binding sites that can accommodate increasing dye loading without significant saturation effects.

The $\pi$-$\pi$ stacking analysis, as depicted in Fig. 7(b), offers crucial mechanistic insights into the adsorption preference and behavior of SSY dye molecules on hydrochar surfaces. The number of SSY-HC1 $\pi$-$\pi$ interactions systematically increases with increasing SSY concentration, rising from less than 1 interaction for 10 SSY molecules to over 80 interactions for 300 SSY molecules. This observation highlights that hydrochar surfaces provide increasingly favorable sites for aromatic stacking as dye loading increases, underscoring the importance of $\pi$-$\pi$ interactions in mediating the adsorption process and driving preferential dye adsorption onto hydrochar surfaces. In contrast, SSY-SSY $\pi$-$\pi$ interactions remain relatively low at low concentrations (< 100 SSY), indicating minimal dye self-aggregation. However, as dye concentration surpasses 100 SSY molecules, the incidence of SSY-SSY $\pi$-$\pi$ interactions increases significantly, suggesting that higher dye concentrations promote dye self-aggregation and potentially compete with hydrochar for favorable interaction sites. This finding emphasizes the importance of optimizing dye concentration to maximize adsorption efficiency while minimizing self-aggregation phenomena.

The dramatic difference in interaction preferences can be attributed to several molecular-level factors. First, the extended aromatic structure of hydrochar provides larger and more stable $\pi$-electron systems compared to individual dye molecules, enabling stronger $\pi$-$\pi$ stacking interactions. Second, the framework of hydrochar presents optimally oriented aromatic surfaces that complement the planar structure of SSY molecules.

The interaction provides valuable insights into the concentration-dependent adsorption mechanism. To quantify this relationship, we performed linear fitting using the equation $y = a \times x$, where x represents the number of SSY molecules in the system, and y denotes the number of van der Waals contacts or $\pi$-$\pi$ interactions. Notably, both van der Waals contacts and $\pi$-$\pi$ stacking interactions exhibit excellent linear correlations with SSY concentration, as evidenced by high correlation coefficients (R$^{2}$) of 0.956 and 0.984, respectively. This systematic scaling demonstrates that each additional dye molecule contributes proportionally to the total interaction capacity across the entire concentration range, indicating efficient utilization of available binding sites and highlighting the concentration-dependent nature of the adsorption process.

### Functional group effects

**Fig. 8:**
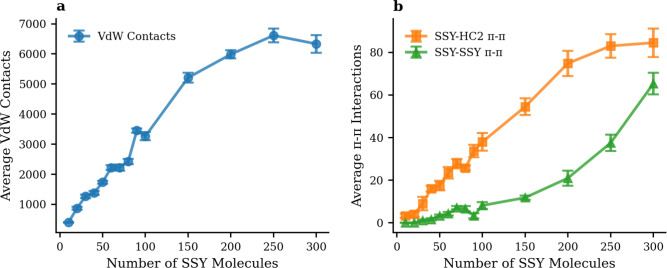
Concentration-dependent molecular interactions between SSY dye molecules and HC2 hydrochar across different SSY loading systems. (**a**) Average van der Waals contacts. (**b**) Average $\pi$-$\pi$ stacking interactions.

**Fig. 9:**
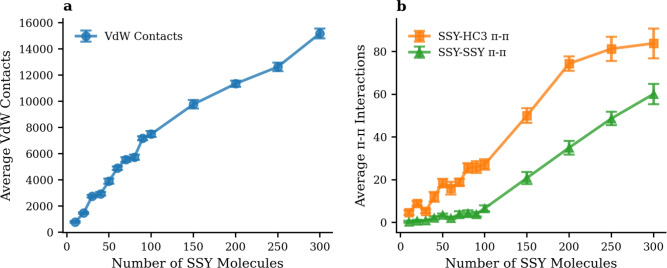
Concentration-dependent molecular interactions between SSY dye molecules and HC3 hydrochar across different SSY loading systems. (**a**) Average van der Waals contacts. (**b**) Average $\pi$-$\pi$ stacking interactions.

Figures 8 and 9 present interaction analyses for HC2 and HC3 systems, respectively, enabling direct comparison with the HC1 results presented in Fig. 7.

**Table 3:** Molecular interaction and linear fitting for three hydrochar architectures.

**Linear relation**$y = a\times x$	**HC1**	**HC2**	**HC3**
Van der Waals Interactions			
Linear Coefficient (a)	40.3	22.9	47.7
Correlation (R$^{2}$)	0.956	0.927	0.962
$\pi$-$\pi$ Stacking Interactions			
SSY-Hydrochar Linear Coefficient (a)	0.290	0.310	0.311
SSY-Hydrochar Correlation (R$^{2}$)	0.984	0.966	0.968
SSY-SSY Linear Coefficient (a)	0.219	0.192	0.217
SSY-SSY Correlation (R$^{2}$)	0.939	0.876	0.953

The data reveals several critical insights into functional group effects. HC3 exhibits the highest VdW contact capacity, scaling from 777 contacts at 10 SSY to 15,196 contacts at 300 SSY, representing a 19.6-fold increase. This superior performance can be attributed to the enhanced surface polarity and hydrogen bonding capacity of hydroxyl groups, which create more favorable interaction sites for dye molecules. HC1 shows intermediate performance with a 16.7-fold increase (from 802 to 13,391 contacts), while HC2 exhibits the lowest capacity with a 16.0-fold increase (from 395 to 6,334 contacts).

The comparison of HC1, HC2, and HC3 systems reveals distinct interaction patterns that directly correlate with their surface chemistry and functional group composition. The linear scaling behavior of molecular interactions provides quantitative insights into the adsorption efficiency of each system. Table 3 presents linear fitting coefficients, correlation metrics, interaction ranges, and system characteristics for all three hydrochar architectures, providing complete evidence of functional group effects on molecular interactions.

Table 3 provides quantitative insights into the scaling behavior of interactions with SSY concentration across all three hydrochar systems. For van der Waals interactions, HC3 exhibits the highest linear coefficient (47.7 interactions per SSY molecule), followed by HC1 with 40.3, and HC2 with 22.9. This ranking directly reflects the functional group density, with HC3 providing the most efficient VdW interaction scaling. The $\pi$-$\pi$ stacking interactions show remarkably similar linear coefficients across all three systems (0.290–0.311.290.311), indicating that aromatic stacking efficiency is largely independent of surface functionalization. However, HC3 maintains the highest $\pi$-$\pi$ HC coefficient (0.311), suggesting that hydroxyl groups enhance both VdW and $\pi$-$\pi$ interaction scaling. The dye-dye self-interaction coefficients are consistent across systems (0.192–0.217.192.217), indicating that dye aggregation behavior is not significantly influenced by hydrochar surface chemistry. These linear relationships confirm the systematic and predictable nature of the adsorption process across all three architectures, supporting their use as reliable adsorbent materials across a wide range of operating conditions.

All three systems demonstrate excellent $\pi$-$\pi$ interaction correlations (R$^{2}>$ 0.96), indicating that aromatic stacking is a fundamental mechanism regardless of functional group composition. However, HC1 achieves the highest correlation coefficient (R$^{2}$ = 0.984), suggesting that mixed functionalization provides optimal $\pi$-electron system organization. HC2 and HC3 show comparable $\pi$-$\pi$ performance (R$^{2}$ = 0.966 and 0.968, respectively), indicating that both carbon-only and hydroxyl-rich architectures can effectively support aromatic interactions.

The systematic variation in performance across the three architectures demonstrates that surface chemistry can be strategically tuned to optimize specific interaction types. Hydroxyl functionalization enhances VdW interactions through increased polarity and hydrogen bonding capacity, while mixed functionalization (HC1) provides balanced performance across multiple interaction mechanisms.

### Adsorption isotherms

**Fig. 10:**
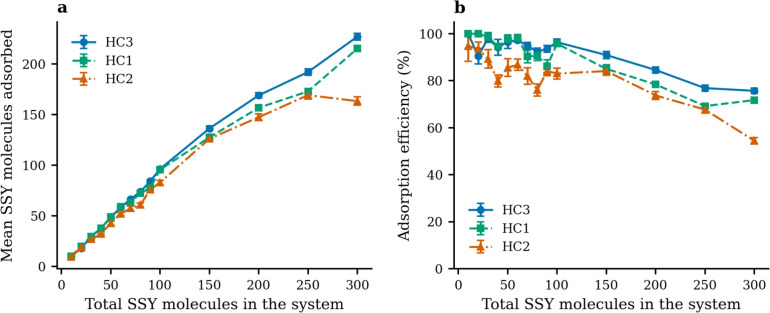
SSY molecule adsorption as a function of total number of SSY molecules in the system for three hydrochar models. (Left) Mean number of SSY molecules adsorbed. (Right) Adsorption efficiency (%).

This section systematically analyzes Sunset Yellow adsorption on three hydrochar models to evaluate the impact of surface functionalization on dye removal efficiency. Figure 10 compares HC1, HC2, and HC3 models using capacity (mean SSY adsorbed) and adsorption efficiency (percentage of SSY adsorbed). The results reveal that at low total SSY concentrations (less than 100), hydrochar effectively adsorbs nearly all the dye molecules. However, as the total SSY concentration increases, a portion of the dye remains free in the water medium or self-aggregates, indicating potential saturation limits for efficient dye removal.

Across all loadings, HC3 exhibits the highest adsorption capacity and efficiency, followed by HC1, with HC2 showing the lowest values. At representative intermediate loadings (e.g., 100–200 SSY), HC3 approaches near-complete adsorption (efficiency $\gtrsim 95\%$), while HC1 reaches high but slightly lower levels, and HC2 lags behind by a substantial margin. These trends suggest that hydroxyl functionalization enhances SSY–surface interactions, likely by increasing the density and strength of favorable interaction sites, thereby promoting adsorption.

The monotonic increase of the mean number adsorbed with SSY loading is consistent with a capacity-limited adsorption process; however, the efficiency panel reveals diminishing returns at the highest loadings for HC1 and especially HC2, indicative of competition for adsorption sites and increased steric crowding. In contrast, HC3 sustains a higher efficiency over the same loading window, pointing to more effective utilization of adsorption sites and/or improved SSY affinity.

**Fig. 11:**
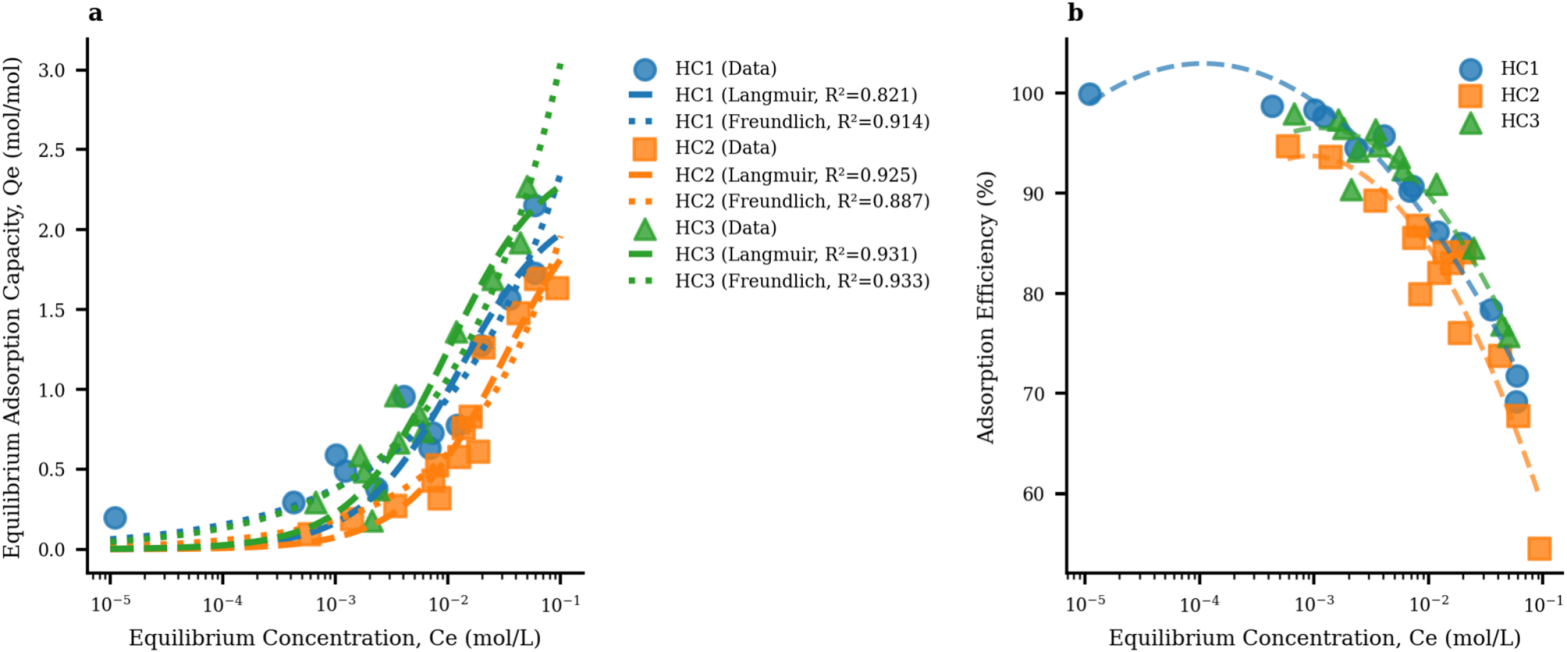
Adsorption isotherm for all three hydrochar systems. (**a**) Adsorption isotherms (Qe vs Ce) showing equilibrium adsorption capacity as a function of equilibrium concentration, with Langmuir and Freundlich model fits. (**b**) Adsorption efficiency vs equilibrium concentration revealing concentration-dependent performance trends.

The adsorption isotherms across all three hydrochar systems offer quantitative insights into the concentration-dependent adsorption behavior, enabling a comprehensive comparison of performance characteristics among HC1, HC2, and HC3 models. Figure 11 showcases adsorption isotherm in the form of efficiency-concentration relationships, which visually depict how the adsorption efficiency varies with the concentration of Sunset Yellow (SSY) dye molecules in the system. By examining these relationships, one can gain a deeper understanding of the adsorption mechanisms and identify the hydrochar model that exhibits optimal performance for SSY removal under various concentrations. This information is crucial for guiding the selection and design of efficient adsorbent materials tailored to specific dye removal applications.

**Table 4:** Adsorption isotherm model fitting parameters for all three hydrochar systems.

**System**	**Model**	${\textbf {Q}}_{{\textbf {max}}}$ **(mol/mol)**	${\textbf {K}}_{{\textbf {L}}} {\textbf {(L/mol)}}$	${\textbf {K}}_{{\textbf {F}}}$	**n**	**R**$^{{\textbf {2}}}$
HC1	Langmuir	2.219	80.82	–	–	0.821
	Freundlich	–	–	5.838	2.529	0.914
HC2	Langmuir	2.382	32.01	–	–	0.925
	Freundlich	–	–	6.408	1.944	0.887
HC3	Langmuir	2.505	98.92	–	–	0.931
	Freundlich	–	–	8.637	2.205	0.933

**Table 5:** Performance comparison across all hydrochar systems.

**Metric**	**HC1**	**HC2**	**HC3**
**Average Efficiency (%)**	89.7 ± 10.4	81.1 ± 10.6	91.5 ± 7.5
**Maximum**${\textbf {Q}}_{{\textbf {e}}}$ **(mol/mol/mg/g)**	2.153/544.8	1.693/442.6	2.271/483.8
**Low Conc**${\textbf {Q}}_{{\textbf {e}}}$ **(mol/mol/mg/g)**	0.292/73.9	0.259/67.7	0.287/61.1
**High Conc**${\textbf {Q}}_{{\textbf {e}}}$ **(mol/mol/mg/g)**	1.817/459.8	1.601/418.5	1.961/417.7

The adsorption efficiency at different SSY loading (Fig. 11b) reveals critical insights into the stability of adsorption performance across different operating conditions. HC3 demonstrates the most stable efficiency profile, with efficiency decreasing from 95.5% at low concentrations to 82.0% at high concentrations ($\Delta$ = −13.5%). In contrast, HC1 shows the most dramatic efficiency decline, from 98.7% to 76.1% ($\Delta$ = −22.6%), while HC2 exhibits moderate sensitivity with efficiency dropping from 90.0% to 71.2% ($\Delta$ = −18.8%).

This concentration-dependent behavior can be attributed to several factors. As concentration increases, available adsorption sites become limited due to site saturation, leading to reduced efficiency. Higher dye concentrations also increase competition for binding sites, particularly affecting systems with limited functional group diversity. Additionally, increased molecular crowding at high concentrations can reduce the effective surface area available for adsorption through steric effects.

The adsorption data were fitted to both Langmuir and Freundlich isotherm models to understand the underlying adsorption mechanisms and quantify the thermodynamic parameters. Table 4 presents the fitted parameters for all three systems, revealing the fundamental differences in adsorption behavior.

The Langmuir model fitting results show that HC3 achieves the highest maximum adsorption capacity (Qmax = 2.505 mol/mol) and the highest Langmuir constant ($K_L$ = 98.92 L/mol). HC2 shows the lowest $K_L$ value (32.01 L/mol), suggesting reduced interaction strength with the carbon-only surface. The Freundlich model fitting reveals that HC3 also has the highest $K_F$ value (8.637), confirming its superior adsorption capacity across all concentration ranges. Based on the isotherms, the three hydrochar systems can be ranked according to different performance criteria. HC3 demonstrates the highest capacity (2.271 mol/mol), most stable efficiency (91.5% ± 7.5%), and best Langmuir fit (R$^{2}$ = 0.931), making it the top performer overall. HC1 shows balanced performance (2.153 mol/mol) with good low-concentration efficiency and the best Freundlich fit (R$^{2}$ = 0.914), while HC2 exhibits lower capacity (1.693 mol/mol), reduced efficiency stability, and moderate model fitting (R$^{2}$ = 0.925).

The results demonstrate that functional group engineering significantly impacts both adsorption capacity and efficiency stability. Hydroxyl functionalization provides the most robust performance across all concentration ranges, while mixed functionalization offers balanced characteristics suitable for diverse applications. These findings provide quantitative guidance for the rational design of hydrochar materials optimized for specific water treatment applications.

### Discussion

The performance reveals distinct advantages for each hydrochar architecture across different application scenarios. HC3 demonstrates superior performance in high-capacity applications with the highest maximum adsorption capacity (2.271 mol/mol) and most stable efficiency profile (88.8% average). HC1 excels in low-concentration applications with the highest efficiency at low concentrations (98.7%) and provides the most balanced performance across all metrics. HC2 offers cost-effective solutions with moderate performance but requires higher adsorbent quantities for equivalent removal.

The molecular interaction shows that HC3 achieves the highest van der Waals interaction coefficient (47.7), indicating superior physical adsorption capacity, while all three systems demonstrate excellent $\pi$-$\pi$ stacking correlations, confirming the fundamental role of aromatic interactions in dye adsorption. The molecular interaction also reveals that $\pi$-$\pi$ stacking interactions between aromatic dye molecules and hydrochar surface constitute the primary adsorption mechanism. The preference for SSY-HC over SSY-SSY $\pi$-$\pi$ interactions can be attributed to several factors. First, the extended aromatic structure of hydrochar provides larger and more stable $\pi$-electron systems compared to individual dye molecules, enabling stronger $\pi$-$\pi$ stacking interactions. Second, the framework of hydrochar presents optimally oriented aromatic surfaces that complement the planar structure of SSY molecules. Third, the presence of functional groups on hydrochar surfaces can enhance $\pi$-$\pi$ interactions through additional stabilizing forces such as hydrogen bonding and electrostatic interactions.

This molecular-level understanding has important implications for the design of enhanced adsorbent materials. The dominance of SSY-HC $\pi$-$\pi$ interactions suggests that increasing the aromatic content and optimizing the surface orientation of hydrochar could further enhance dye removal efficiency. Additionally, the relatively low SSY-SSY interaction tendency indicates that the system is less prone to dye aggregation that could potentially reduce adsorption capacity at higher concentrations.

The molecular dynamics simulations in this work successfully capture the key features of experimental adsorption behavior, including the rapid initial adsorption kinetics and high removal efficiencies observed in laboratory studies. The predicted adsorption capacities and efficiency trends align well with experimental observations, validating the molecular models and simulation methodology employed in this study.

## Conclusions

This study has demonstrated the effective use of molecular dynamics simulations to investigate the adsorption behavior of Sunset Yellow (SSY) dye on three distinct hydrochar architectures, providing molecular-level insights into the role of surface functional groups in dye removal. The systematic comparison revealed that surface chemistry critically determines adsorption capacity and efficiency. The hydroxyl-functionalized hydrochar (HC3) showed the highest adsorption capacity (2.27 mol/mol) and most stable efficiency profile (91.5%), representing a 35% enhancement over the non-functionalized structure (HC2). This establishes a clear correlation between functional group density and adsorption performance, emphasizing the importance of surface functionalization in hydrochar design.

Adsorption was found to occur through a cooperative mechanism involving van der Waals interactions, $\pi$–$\pi$ stacking, and hydrogen bonding. Hydroxyl groups increase local polarity and hydrogen-bonding capacity, enhancing dye accessibility and contact density while preserving strong $\pi$–$\pi$ stacking on aromatic domains. The observed SSY–SSY stacking at high concentrations further contributes to the overall adsorption efficiency and highlights the unique self-assembly behavior of SSY molecules.

These findings provide quantitative and mechanistic guidance for the design of efficient hydrochar-based adsorbents. Hydroxyl-rich architectures are optimal for high-capacity dye removal, while mixed-functional systems (HC1) offer balanced and stable performance. Future studies should consider temperature, pH, and ionic strength effects and validate these mechanisms experimentally. Overall, hydroxyl functionalization represents a powerful approach for enhancing hydrochar adsorption efficiency and advancing sustainable water treatment technologies.

## Data Availability

The datasets generated and/or analyzed during the current study are available from the corresponding author on reasonable request.
